# Supplementary material for: Quantum physics in connected worlds
Source: Nat Commun. 2022 Dec 2;13:7445. doi: 10.1038/s41467-022-35090-y (PMC9718787; doi:10.1038/s41467-022-35090-y)
Supplement: Supplementary file 1 — Supplementary Information [file 41467_2022_35090_MOESM1_ESM.pdf]

Supplementary Information for Quantum physics in Connected Worlds

Joseph Tindall[1,2], Amy Searle[2], Abdulla Alhajri[2,3], and Dieter Jaksch[4,5,2]

[1]Center for Computational Quantum Physics, Flatiron Institute, New York
[2]Department of Physics, University of Oxford
[3]Technology Innovation Institute, Abu Dhabi, UAE
[4]The Hamburg Centre for Ultrafast Imaging, Hamburg, Germany
[5]Institut für Laserphysik, Universität Hamburg, Hamburg, Germany

November 29, 2022

1 Proof of the Equivalence of the XYZ Free Energy on the Complete and Erdős-Rényi (ER) Graphs

We redefine, for clarity, the Hamiltonian from the main text

$$\hat{H}(\mathcal{G}) = \frac{L}{N_E}\left(\sum_{(v,v')\in E}\hat{h}_{v,v'}\right) + \sum_{v\in V}\hat{h}_v, \tag{S1}$$

with

$$\begin{aligned}\hat{h}_{v,v'} &= J_x\hat{s}^x_v\hat{s}^x_{v'} + J_y\hat{s}^y_v\hat{s}^y_{v'} + J_z\hat{s}^z_v\hat{s}^z_{v'}, \qquad J_x, J_y, J_z \in \mathbb{R},\\ \hat{h}_v &= w_x\hat{s}^x_v + w_y\hat{s}^y_v + w_z\hat{s}^z_v, \qquad w_x, w_y, w_z \in \mathbb{R}.\end{aligned} \tag{S2}$$

All quantities retain their meaning from the main text. We also reintroduce the free energy density of a matrix of size $d^L \times d^L$ as

$$f(\hat{A}) = -\frac{1}{L\beta}\ln\left(\mathrm{Tr}(e^{-\beta\hat{A}})\right), \qquad \beta \in \mathbb{R}_{\geq 0}. \tag{S3}$$

In this section we prove the following theorem from the main text:

Theorem 1 *Let $\mathcal{G}_{\mathrm{ER}}(p)$ be an instance of the Erdős-Rényi graph with finite edge probability $0 < p \leq 1$ and L vertices. Let $\mathcal{G}_{\mathrm{Complete}}$ be the complete graph over L vertices. Then, given an arbitrary set of values for the microscopic parameters $\{J_x, J_y, J_z, w_x, w_y, w_z\}$,*

$$\lim_{L\to\infty} f(\hat{H}(\mathcal{G}_{\mathrm{ER}}(p))) = \lim_{L\to\infty} f(\hat{H}(\mathcal{G}_{\mathrm{Complete}})) \qquad \forall\beta \in \mathbb{R}_{\geq 0}. \tag{S4}$$

Moreover, for finite L, we have $|f(\hat{H}(\mathcal{G}_{\mathrm{ER}}(p))) - f(\hat{H}(\mathcal{G}_{\mathrm{Complete}}))| = \mathcal{O}(L^{-1/2})$.

Theorem 1 implies the equivalence, at any temperature, between the equilibrium states (with trace unity) $\rho(\mathcal{G}_{\text{ER}}(p)) \propto \exp(-\beta \hat{H}(\mathcal{G}_{\text{ER}}(p)))$ and $\rho(\mathcal{G}_{\text{Complete}}) \propto \exp(-\beta \hat{H}(\mathcal{G}_{\text{Complete}}))$ in terms of any thermodynamic quantity which can be written as a function of the free energy density. Such quantities dictate the macroscopic phase of the system and are thus of great import. We do not, however, make statements about out-of-equilibrium scenarios or purely local observables which cannot be derived from the free energy density — these are beyond the scope of this paper and an interesting area of future study.

As a high level sketch of the proof: We will prove a Lemma on a pair of Hermitian matrices $\hat{A}$ and $\hat{B}$ which dictates that the difference in their free energy densities can be bounded in terms of the largest (by magnitude) eigenvalue of $\hat{A} - \hat{B}$. We will then, first for spin $s = 1/2$ and then generally for finite s, explicitly identify the scaling on the largest eigenvalue (by magnitude) of $\hat{H}(\mathcal{G}_{\text{ER}}(p)) - \hat{H}(\mathcal{G}_{\text{Complete}})$ and invoke the aforementioned Lemma.

The Lemma that we will use to help prove Theorem 1 is as follows

Lemma 2 *Let $\hat{A}_1, \hat{A}_2, ...$ and $\hat{B}_1, \hat{B}_2, ...$ be two sequences of Hermitian matrices. The matrices A_L and B_L in the sequence have size $d^L \times d^L$ – with d fixed. Let $\hat{D}_L = \hat{A}_L - \hat{B}_L$ and λ^D_{Max} be the largest (in terms of absolute value) eigenvalue of $\hat{D}_L$. If $|\lambda^D_{\text{Max}}| = \mathcal{O}(L^\gamma)$ then $|f(\hat{A}_L) - f(\hat{B}_L)| = \mathcal{O}(L^{\gamma-1})$, which vanishes for $\gamma < 1$ as $L \to \infty$.*

We proceed to prove this Lemma, dropping the subscript on $\hat{A}_L$ and $\hat{B}_L$ – from here on it is always implied that they are of size $d^L \times d^L$. First, order the eigenvalues of $\hat{A}$, $\hat{B}$ and $\hat{D}$ as $\lambda^\eta_1, \lambda^\eta_2, ..., \lambda^\eta_{d^L}$, with $\lambda^\eta_i \geq \lambda^\eta_{i-1}$ and $\eta = \hat{A}, \hat{B}$ or $\hat{D}$. By Weyl's inequality we have $\lambda^B_i + \lambda^D_1 \leq \lambda^A_i \leq \lambda^B_i + \lambda^D_{d^L}$. Now, it follows from this inequality and $|\lambda^D_{\text{Max}}| = \mathcal{O}(L^\gamma)$ that $|\lambda^A_i - \lambda^B_i| = \mathcal{O}(L^\gamma)\ \forall i$. We can therefore write $\lambda^A_i = \lambda^B_i + c_i$ where c_i is a real number such that $|c_i| = \mathcal{O}(L^\gamma)$.

Now define the absolute free energy density difference of $\hat{A}$ and $\hat{B}$, substituting in $\lambda^A_i = \lambda^B_i + c_i$:

$$\Delta f = |f(\hat{A}) - f(\hat{B})| = \Big| \frac{1}{L} \ln \left(\frac{\text{Tr}(\exp(-\beta \hat{A}))}{\text{Tr}(\exp(-\beta \hat{B}))} \right) \Big| = \Big| \frac{1}{L} \ln \left(\frac{\sum_{i=1}^{d^L} \exp(-\beta(c_i)) \exp(-\beta(\lambda^A_i))}{\sum_{i=1}^{d^L} \exp(-\beta(\lambda^A_i))} \right) \Big|. \quad \text{(S5)}$$

Define the following vectors of dimension d^L:

$$\begin{aligned} \mathbf{v} &= \left(\exp(-\beta\lambda^A_1/2), \exp(-\beta\lambda^A_2/2), ..., \exp(-\beta\lambda^A_{d^L}/2)\right)^T, \\ \mathbf{c} &= \left(\exp(-\beta c_1), \exp(-\beta c_2), ..., \exp(-\beta c_{d^L})\right)^T, \end{aligned} \quad \text{(S6)}$$

and the matrix $\hat{C} = \text{Diag}(\mathbf{c})$. Observe that

$$\Delta f = \Big| \frac{1}{L} \ln \left(\frac{\langle \mathbf{v}|\hat{C}|\mathbf{v}\rangle}{\langle \mathbf{v}|\mathbf{v}\rangle} \right) \Big| = \Big| \frac{1}{L} \ln \left(\langle \tilde{\mathbf{v}}|\hat{C}|\tilde{\mathbf{v}}\rangle \right) \Big|, \quad \text{(S7)}$$

where we have defined $\tilde{\mathbf{v}} = \mathbf{v}/\sqrt{\langle \mathbf{v}|\mathbf{v}\rangle}$. As $\langle \tilde{\mathbf{v}}|\tilde{\mathbf{v}}\rangle = 1$, the argument of the logarithm is clearly bounded by the largest eigenvalue (by magnitude) λ^C_{Max} of $\hat{C}$. From $|c_i| = \mathcal{O}(L^\gamma)\ \forall i$ we have $|\lambda^C_{\text{Max}}| = \exp(\mathcal{O}(L^\gamma))$ and thus

$$\Delta f = \Big| \frac{1}{L} \ln \left(\exp(\mathcal{O}(L^\gamma)) \right) \Big| = \mathcal{O}(L^{\gamma-1}), \quad \text{(S8)}$$

completing our proof of Lemma 2 (the sign on the argument of the exponential $\exp(\mathcal{O}(L^\gamma))$ is not of concern as we are taking the absolute value of its logarithm).

Now that we have proven Lemma 2 we will proceed to show that the largest magnitude eigenvalue of the difference matrix $\hat{H}(\mathcal{G}_{\text{ER}}(p)) - \hat{H}(\mathcal{G}_{\text{Complete}})$ scales as $\mathcal{O}(L^{1/2})$ with system size L. This will allow us to utilise Lemma 2 with $\gamma = 1/2$ to prove Theorem 1.

First, let us write $\hat{H}(\mathcal{G}_{\mathrm{ER}}(p))$ as $\hat{H}(\mathcal{G}) = L\sum_{\alpha} J_{\alpha} \cdot \hat{O}^{\alpha}(\mathcal{G}) + \hat{C}$ where $\hat{C}$ is a graph independent operator corresponding to the single-site terms and $\hat{O}^{\alpha}(\mathcal{G})$ is defined as follows.

$$\hat{O}^{\alpha}(\mathcal{G}) = \frac{1}{N_E} \sum_{(v,v')\in E} \hat{s}^{\alpha}_{v} \hat{s}^{\alpha}_{v'}. \tag{S9}$$

We now provide the following Lemma involving $\hat{O}^{\alpha}(\mathcal{G})$

Lemma 3 *Let L be the number of vertices in $\mathcal{G}_{\mathrm{ER}}(p)$ and $\mathcal{G}_{\mathrm{Complete}}$. Let $|\psi\rangle$ be any state such that $\langle\psi|\psi\rangle = 1$. Then $\left|\langle\psi|\hat{O}^{\alpha}(\mathcal{G}_{\mathrm{ER}}(p))|\psi\rangle - \langle\psi|\hat{O}^{\alpha}(\mathcal{G}_{\mathrm{Complete}})|\psi\rangle\right| = \mathcal{O}(L^{-1/2})$.*

If we can prove this Lemma then, as $\hat{H}(\mathcal{G}_{\mathrm{ER}}(p)) - \hat{H}(\mathcal{G}_{\mathrm{Complete}}) = L\sum_{\alpha} J_{\alpha}(\hat{O}^{\alpha}(\mathcal{G}_{\mathrm{ER}}(p)) - \hat{O}^{\alpha}(\mathcal{G}_{\mathrm{Complete}}))$, the largest magnitude eigenvalue of the difference matrix $\hat{H}(\mathcal{G}_{\mathrm{ER}}(p)) - \hat{H}(\mathcal{G}_{\mathrm{Complete}})$ will be bounded as $\mathcal{O}(L^{1/2})$, letting us invoke Lemma 2 to prove Theorem 1 as desired.

1.1 Proof of Lemma 3 for Spin 1/2

We set $\hat{s}^{\alpha}_{v} = \frac{1}{2}\hat{\sigma}^{\alpha}_{v}$, where $\hat{\sigma}^{\alpha}_{v}$ is the Pauli matrix for spin $\alpha = x, y$ or z on vertex V. The dimension of the Hilbert space for a given L is thus 2^L. In this context, the two-body operator $\hat{O}^{\alpha}(\mathcal{G})$ reads (we have dropped the factor of s^2 as it is inconsequential)

$$\hat{O}^{\alpha}(\mathcal{G}) = \frac{1}{N_E} \sum_{(v,v')\in E} \hat{\sigma}^{\alpha}_{v} \hat{\sigma}^{\alpha}_{v'}. \tag{S10}$$

In order to prove Lemma 3 we choose to work in the eigenbasis $|\sigma^{\alpha}_{1}, ..., \sigma^{\alpha}_{L}\rangle$ which diagonalises both $\hat{O}^{\alpha}(\mathcal{G}_{\mathrm{ER}}(p))$ and $\hat{O}^{\alpha}(\mathcal{G}_{\mathrm{Complete}})$, with $\sigma^{\alpha}_{v} = \pm 1$. The eigenvalues for $\hat{O}^{\alpha}(\mathcal{G}_{\mathrm{Complete}})$ are straightforward as we can define the 'total spin' scalar $M_{\alpha} = \sum_{v} \sigma^{\alpha}_{v}$ to see that

$$\langle \sigma^{\alpha}_{1}, ..., \sigma^{\alpha}_{L}|\hat{O}^{\alpha}(\mathcal{G}_{\mathrm{Complete}})|\sigma^{\alpha}_{1}, ..., \sigma^{\alpha}_{L}\rangle = \frac{1}{L(L-1)}\left((M_{\alpha})^2 - L\right) = \left(\frac{M_{\alpha}}{L}\right)^2 + \mathcal{O}(L^{-1}), \tag{S11}$$

where we have used the fact $\sigma^{\alpha}_{v}\sigma^{\alpha}_{v} \equiv 1$ and $N_E = \frac{1}{2}L(L-1)$ for the complete graph.

For $\hat{O}^{\alpha}(\mathcal{G}_{\mathrm{ER}}(p))$ we will utilise the notion of graph cuts to determine the corresponding eigenvalue of the state $|\sigma^{\alpha}_{1}, ..., \sigma^{\alpha}_{L}\rangle$. Specifically, this state can be considered a bi-partition of the underlying Erdős-Rényi graph with the vertices for which $\sigma^{\alpha}_{v} = 1$ corresponding to one set (A) and the vertices where $\sigma^{\alpha}_{v} = -1$ being the other (B). The partition sizes are $(L + M_{\alpha})/2$ and $(L - M_{\alpha})/2$ respectively, with $M_{\alpha} = \sum_{v} \sigma^{\alpha}_{v}$.

We then have

$$\langle \sigma^{\alpha}_{1}, ..., \sigma^{\alpha}_{L}|\hat{O}^{\alpha}(\mathcal{G}_{\mathrm{ER}}(p))|\sigma^{\alpha}_{1}, ..., \sigma^{\alpha}_{L}\rangle = N_E(\mathcal{G}_{\mathrm{ER}}(p)) - 2N_{AB}, \tag{S12}$$

where $N_E(\mathcal{G}_{\mathrm{ER}}(p))$ is the number of edges associated with the given ER graph, N_{AB} is the number of edges between the sets and is often referred to the 'cut-size' of the partition.

Because all of the sites and edges in an Erdős-Rényi graph are independent a central limit theorem applies [1, 2] and the cut-size N_{AB} for a randomly selected state $|\sigma^{\alpha}_{1}, ..., \sigma^{\alpha}_{L}\rangle$ will be a binomially distributed random variable with number of trials $n = (L^2 - M^2_{\alpha})/4$ and probability p, i.e. $N_{AB} \sim B((L^2 - M^2_{\alpha})/4, p)$. The mean μ of this Binomial distribution is $p(L^2 - M^2_{\alpha})/4$ and the standard deviation is $\sqrt{p(1-p)(L^2 - M^2_{\alpha})}/2$. We wish to quantify the most extreme cuts which can occur with non-vanishing probability and thus place strict bounds on the deviation of N_{AB} from its mean.

Such extremes can be found by applying a Chernoff bound [3] on the tails of the Binomial distribution. Importantly, we then need to take a union bound over all cuts because we wish to ensure that this bound is tight for all 2^L states and thus valid for the whole spectrum of $\hat{O}^\alpha(\mathcal{G}_{\rm ER}(p))$.

To make the application of these bounds explicit let us consider the case when $M_\alpha = 0$. Following the Chernoff bound we have the probability that a given cut size N_{AB} exceeds the mean μ by a factor of δ is bounded via

$$P(N_{AB} \geq (1+\delta)\mu) \leq e^{-\delta^2\mu/3}, \tag{S13}$$

with $0 \leq \delta \leq 1$. We need, however, to ensure that this bound is tight for all $\binom{L}{L/2}$ cuts where $M_\alpha = 0$. Thus we can apply the union bound to get

$$P'(N_{AB} \geq (1+\delta)\mu) \leq \binom{L}{L/2} e^{-\delta^2\mu/3}, \tag{S14}$$

where $P'(N_{AB} \geq (1+\delta)\mu)$ is the probability that, of all the cuts with $M_\alpha = 0$, at least one of them deviates exceeds the mean by a factor of δ. For large L we can use a continuity approximation on the Binomial coefficient and reintroduce $\mu = p(L^2 - M_\alpha^2)/4$ to get

$$P'(N_{AB} \geq (1+\delta)p(L^2 - M_\alpha^2)/4) \leq \sqrt{\frac{2}{\pi L}} e^{L\log_e(2) - p\delta^2 L^2/12}. \tag{S15}$$

It is clear this probability vanishes unless $\delta \leq O(L^{-1/2})$. Therefore N_{AB} can never exceed the mean by more than $O(L^{3/2})$ which vanishes in comparison to the mean. An identical result can be obtained for the lower end of the Binomial distribution. A similar result can also be obtained for any value of M and we can apply the union bound over all possible M values without weakening the result due to the strength of e^L in comparison to a sum which only extends linearly in L.

Further details on bounding the spectrum of cuts in ER graphs can be found in Refs. [1, 2]. Formally, we can state that for absolutely *any* state $|\sigma_1^\alpha, ..., \sigma_L^\alpha\rangle$

$$|N_{AB} - \frac{1}{4}p(L^2 - M_\alpha^2)| = \mathcal{O}(L^{3/2}). \tag{S16}$$

It therefore follows that $\lim_{L\to\infty} N_{AB}/L^2 = \frac{1}{4}p(L^2 - M_\alpha^2)$. We also know that $N_E(\mathcal{G}_{\rm ER}(p)) = \frac{1}{2}pL^2 + \mathcal{O}(L)$. Combining these results together we find that for absolutely *any* state $|\sigma_1^\alpha, ..., \sigma_L^\alpha\rangle$

$$\langle\sigma_1^\alpha, ..., \sigma_L^\alpha|\hat{O}^\alpha(\mathcal{G}_{\rm ER}(p))|\sigma_1^\alpha, ..., \sigma_L^\alpha\rangle = \frac{1}{N_E(\mathcal{G}_{\rm ER}(p))}(N_E(\mathcal{G}_{\rm ER}(p)) - 2N_{AB}) = \left(\frac{M_\alpha}{L}\right)^2 + \mathcal{O}(L^{-1/2}). \tag{S17}$$

Lemma 3 immediately follows from Eqs. (S11) and (S17) – the proof of Theorem 1 is thus complete for $s = 1/2$

1.2 Spin s

We can extend our proof to all finite spin s. Let us work with the general spin s definition of $\hat{O}^\alpha(\mathcal{G})$:

$$\hat{O}^\alpha(\mathcal{G}) = \frac{1}{N_E} \sum_{(v,v')\in E} \hat{s}_v^\alpha \hat{s}_{v'}^\alpha, \tag{S18}$$

where s_v^α is the canonical spin s operator on vertex V. We work in the basis in which $\hat{O}^\alpha(\mathcal{G})$ is diagonal. The relevant basis states are $|s_1^\alpha, ..., s_L^\alpha\rangle$ with s_v^α taking values in the set $\{-s, -s+1, ..., s\}$.

We now define the scalar $M_\alpha = \sum_v s_v^\alpha$ and obtain

$$\langle s_1^\alpha, ..., s_L^\alpha | \hat{O}^\alpha(\mathcal{G}_{\text{Complete}}) | s_1^\alpha, ..., s_L^\alpha \rangle = \frac{1}{L(L-1)} \left((M_\alpha)^2 - L \right) = \left(\frac{M_\alpha}{L} \right)^2 + \mathcal{O}(L^{-1}), \tag{S19}$$

in direct analogy with the spin 1/2 case. We can also diagonalise $\hat{O}^\alpha(\mathcal{G}_{\text{ER}}(p))$ with the computational basis $|s_1^\alpha, ..., s_L^\alpha\rangle$. A given basis-state represents a partition of the vertices of the graph into $2s+1$ sets, with each set containing vertices which have the same value for s_v^α. We therefore have

$$\langle s_1^\alpha, ..., s_L^\alpha | \hat{O}^\alpha(\mathcal{G}_{\text{Complete}}) | s_1^\alpha, ..., s_L^\alpha \rangle = \frac{1}{N_E(\mathcal{G}_{\text{ER}}(p))} \left(\sum_{i=-s}^{s} i^2 N_E^{(i)} + \sum_{i=-s}^{s} \sum_{j=i+1}^{s} ij N_E^{(i,j)} \right), \tag{S20}$$

where $N_E^{(i)}$ is the number of edges present between all pairs of vertices with the same spin i, with $i \in [-s, -s+1, ..., s]$. Additionally, $N_E^{(i,j)}$ is the number of edges between pairs of vertices where one has spin i and the other spin j.

We now note that each term inside the summations in Eq. (S20) is independent of the other terms due to the independence of edges. We can then, just like for the spin $s = 1/2$ case, invoke the Binomial distribution of edges, both within a set and between any two sets, and apply bounds on the tails of the distribution. From this it then follows that the integers $N_E^{(i)}$ and $N_E^{(i,j)}$ cannot deviate in any significant (i.e. quadratic in L^2) way from their expected values, which are $p(N_i)^2/2$ and pN_iN_j respectively — with N_i the number of vertices with spin i in the state $|s_1^\alpha, ..., s_L^\alpha\rangle$. We also have $N_E(\mathcal{G}_{\text{ER}}(p)) = \frac{1}{2}pL^2 + \mathcal{O}(L)$. We can thus reduce Eq. (S20) to

$$\langle s_1^\alpha, ..., s_L^\alpha | \hat{O}^\alpha(\mathcal{G}_{\text{Complete}}) | s_1^\alpha, ..., s_L^\alpha \rangle = \frac{2}{pL^2} \left(\frac{1}{2} \sum_{i=-s}^{s} i^2 p (N_i)^2 + \sum_{i=-s}^{s} \sum_{j=i+1}^{s} ijpN_iN_j \right) + \mathcal{O}(L^{-1/2}). \tag{S21}$$

Observing that $M^\alpha = \sum_v s_v^\alpha = \sum_{i=-s}^{s} iN_i$ leads us to, for absolutely any basis state $|s_1^\alpha, ..., s_L^\alpha\rangle$,

$$\langle s_1^\alpha, ..., s_L^\alpha | \hat{O}^\alpha(\mathcal{G}_{\text{ER}}(p)) | s_1^\alpha, ..., s_L^\alpha \rangle = \left(\frac{M_\alpha}{L} \right)^2 + \mathcal{O}(L^{-1/2}), \tag{S22}$$

which, when combined with Eq. (S19) gives us proof of Lemma 3. The proof of Theorem 1 is thus complete.

2 Numerical Verification of the Proof

We now provide numerical evidence to support Theorem 1. In order to do so we consider the following matrix

$$\hat{D}(\mathcal{G}_{\text{ER}}(p)) = \hat{H}(\mathcal{G}_{\text{Complete}}) - \hat{H}(\mathcal{G}_{\text{ER}}(p)) = \sum_{\alpha=x,y,z} J_\alpha \sum_{\substack{v,v'=1 \\ v>v'}}^{L} \left(\frac{2}{L-1} - \frac{p_{v,v'} L}{N_E(\mathcal{G}_{\text{ER}}(p))} \right) \hat{\sigma}_v^\alpha \hat{\sigma}_{v'}^\alpha, \tag{S23}$$

which is the difference between $H(\mathcal{G})$ on a given instance $\mathcal{G}_{\text{ER}}(p)$ of the ER graph and $H(\mathcal{G})$ on the complete graph. Here, $p_{v,v'}$ is a random variable which is 1 with probability p and 0 otherwise. The quantity $N_E(\mathcal{G}_{\text{ER}}(p)) = \sum_{v>v'} p_{v,v'}$ is the number of edges of the given ER graph.

To support Theorem 1 we numerically determine the scaling on the largest absolute eigenvalue λ_{Max}^D of $\hat{D}(\mathcal{G}_{\text{ER}}(p))$ as a function of L – for several different choices of the parameter set $\{J_x, J_y, J_z, p\}$. In Supplementary Fig. 1 we plot this scaling and observe a clear linear dependence of λ_{Max}^D on $\sqrt{L}$ – with $\lambda_{\text{Max}}^D/\sqrt{L}$ staying fairly constant with L whilst λ_{Max}^D/L decays. It is this scaling on λ_{Max}^D which we prove in Sections 1.1 and 1.2 and allows us to invoke Lemma 2 to prove Theorem 1.

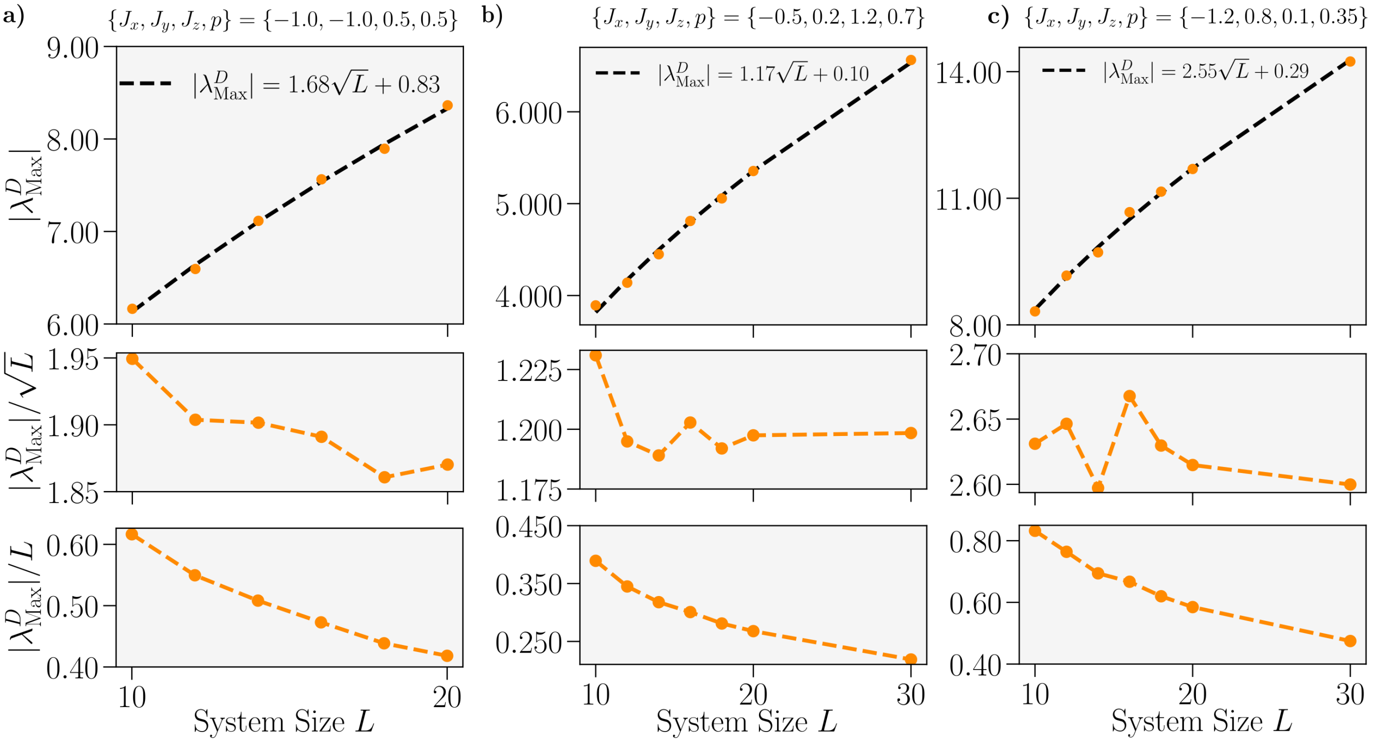

Supplementary Figure 1: Largest eigenvalue (by absolute value) λ^D_{Max} of the difference matrix $\hat{D}(\mathcal{G}_{\mathrm{ER}}(p))$ – see Eq. (S23) – versus system size. a-c) Corresponds to several different sets of the parameters $\{J_x, J_y, J_z, p\}$. Top plots provide the direct scaling of λ^D_{Max} with L whilst the middle and bottom give the scaling of $\lambda^D_{\mathrm{Max}}/\sqrt{L}$ and $\lambda^D_{\mathrm{Max}}/L$ with L respectively. Dashed black line in the top plots represents a fit to the corresponding data – with the explicit equation given in the legend. Each data point corresponds to an average over $n = 100$ draws of the ER graph from its ensemble. System sizes for $L \leq 16$ were obtained by Exact Diagonalisation whilst $L > 16$ were obtained using DMRG on $\pm\hat{D}(\mathcal{G}_{\mathrm{ER}}(p))$ with a bond-dimension $\chi = 10L$.

3 XXZ Hamiltonian on the Complete Graph

We consider the XXZ spin $s = 1/2$ Hamiltonian on the complete graph, dropping the factor of s^2 on the two-body operators as it is irrelevant:

$$\hat{H}_{\mathrm{XXZ}}(\mathcal{G}_{\mathrm{Complete}}) = \frac{2}{L-1} \sum_{\substack{v,v'=1 \\ v>v'}}^{L} -J(\hat{\sigma}^x_v \hat{\sigma}^x_{v'} + \hat{\sigma}^y_v \hat{\sigma}^y_{v'}) + \Delta \hat{\sigma}^z_v \hat{\sigma}^z_{v'}. \tag{S24}$$

We focus on the case where J and Δ are positive, finite, real numbers. In the limit $L \to \infty$ the free energy density for this Hamiltonian is also (by Theorem 1) that for the Hamiltonian $\hat{H}_{\mathrm{XXZ}}(\mathcal{G}_{\mathrm{ER}}(p))$ with finite edge probability p. We can thus focus exclusively on $\hat{H}_{\mathrm{XXZ}}(\mathcal{G}_{\mathrm{Complete}})$ to quantify the equilibrium properties of both cases as $L \to \infty$. We can diagonalise this Hamiltonian by introducing the global operators $\hat{S}^\alpha = \sum_v \hat{\sigma}^\alpha_v$, the Casimir operator $\hat{S}^2 = (\hat{S}^x)^2 + (\hat{S}^y)^2 + (\hat{S}^z)^2$ and the complete total-spin basis $|S, M\rangle$ with $\hat{S}^2|S, M\rangle = 4S(S+1)|S, M\rangle$ and $\hat{S}^z|S, M\rangle = M|S, M\rangle$. The integer S can take values in the range $\{M/2, M/2+1, ..., L/2\}$ whilst M can take values in the range

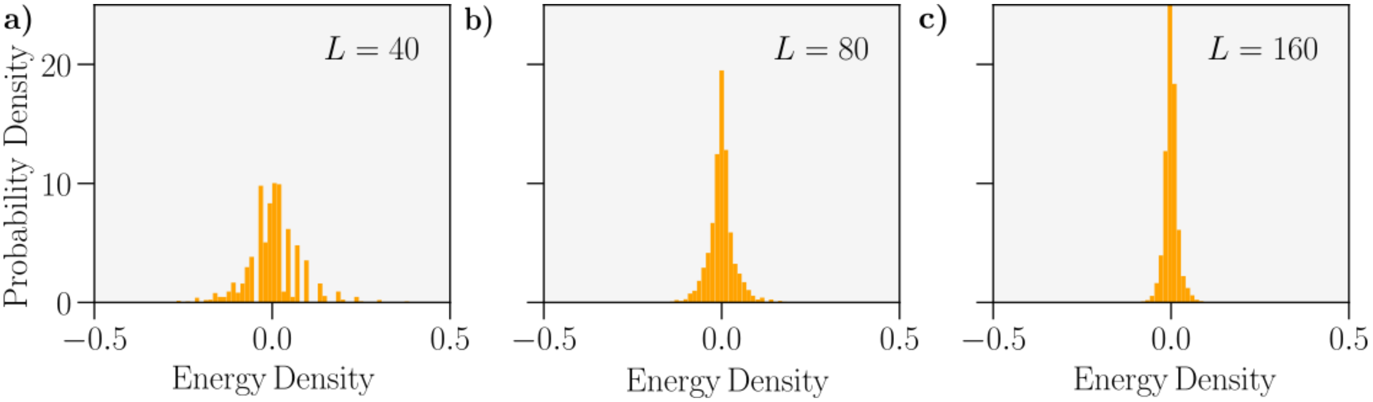

Supplementary Figure 2: Probability Density $p(E)$ for the eigenenergies of $\hat{H}_{\text{XXZ}}(\mathcal{G}_{\text{Comp}})$ – see Eq. (S24) – with $\Delta = 1.5$ and for various system sizes L. The quantity $p(E)dE$ is the probability of a randomly chosen eigenstate having an energy density in the interval $[p(E), p(E+dE)]$. The spectrum was calculated exactly via Eq. (S25) and as $L \to \infty$ tends towards a delta function which also represents the spectrum of $\hat{H}_{\text{XXZ}}(\mathcal{G}_{\text{ER}}(p))$ for any finite non-zero p.

$\{-L, -L+2, ..., L\}$ [4] — assuming L is even. Following a small amount of algebra we have

$$\begin{aligned} &\hat{H}_{\text{XXZ}}(\mathcal{G}_{\text{Complete}})|S, M\rangle = \lambda_{S,M}|S, M\rangle \\ &\lambda_{S,M} = -\frac{J}{L-1}(4S(S+1) - M^2) + \frac{2JL}{L-1} + \frac{\Delta}{L-1}(M^2 - L), \\ &D_{S,M} = \binom{L-1}{L/2 - S - \delta_{S,0}} - \binom{L-1}{L/2 - S - 1 - \delta_{S,0}}, \end{aligned} \tag{S25}$$

where $\delta_{i,j}$ is the Kronecker Delta function and $D_{S,M}$ is the degeneracy of a given $\lambda_{S,M}$ eigenvalue in the full 2^L dimensional Hilbert space. These degeneracies were found via the Clebsch-Gordan coefficients for an SU(2) symmetry in a space formed from an L-fold tensor product of spin 1/2s [5].

Taking the thermodynamic limit we find the spectrum of $\hat{H}_{\text{XXZ}}(\mathcal{G}_{\text{Complete}})$ will thus be dominated by states of 0 energy density as the degeneracy of the $|S, M\rangle$ states is largest when S and M do not grow proportionally with L. In Supplementary Fig. 2 we plot a histogram of this spectrum for a few choice system sizes. The probability density narrows around 0 meaning that the system is dominated by states with 0 energy density.

The ground states meanwhile are far from these 'typical' states and consists of any of the states $|L/2, M\rangle$ where M is finite. More explicitly such states can be written as

$$|\psi\rangle_{\text{GS}} = |L/2, M\rangle \propto \sum_{\sigma_1+\sigma_2+...\sigma_L=M} |\sigma_1, \sigma_2, ..., \sigma_L\rangle, \tag{S26}$$

i.e. they are an equal superposition of all basis states with M total magnetisation.

4 Spin Hamiltonian on an Arbitrary graph with a Non-Trivial Cut

4.1 Proof of Reduction of Free Energy Density to That For Two Large Spins

Here we consider $\hat{H}(\mathcal{G}(\lambda, p_1, p_2))$ where $\hat{H}$ is defined in Eq. (S1) and $\mathcal{G}(\lambda, p_1, p_2)$ is an instance of the random graph with a non-trivial cut and L vertices — as defined in the Methods section of the main

text. We introduce the two sets of vertices $A = \{1, ..., \lambda L\}$ and $B = \{\lambda L + 1, ..., L\}$ and define the following effective Hamiltonian from the parameters λ, p_1 and p_2

$$\hat{H}_{\mathrm{Eff}}(\mathcal{G}(\lambda, p_1, p_2)) = \frac{1}{N} \sum_{\alpha=x,y,z} J_\alpha \left(p_1 (\hat{S}_A^\alpha)^2 + p_1 (\hat{S}_B^\alpha)^2 + 2 p_2 \hat{S}_A^\alpha \hat{S}_B^\alpha \right) + \frac{1}{N} \sum_{\alpha=x,y,z} w_\alpha (\hat{S}_A^\alpha + \hat{S}_B^\alpha), \quad \text{(S27)}$$

where $\hat{S}_A^\alpha = \sum_{v \in A} \hat{s}_v^\alpha$, $\hat{S}_B^\alpha = \sum_{v \in B} \hat{s}_v^\alpha$ and $N = \frac{1}{2} L \left(\lambda^2 p_1 + (\lambda - 1)^2 p_1 + 2\lambda(\lambda - 1) p_2 \right)$.

With these definitions we prove the following theorem

Theorem 4 *For a given set of values for the microscopic parameters* $\{J_x, J_y, J_z, w_x, w_y, w_z\}$ *then*

$$\lim_{L \to \infty} f(\hat{H}(\mathcal{G}(\lambda, p_1, p_2))) = \lim_{L \to \infty} f(\hat{H}_{\mathrm{Eff}}(\mathcal{G}(\lambda, p_1, p_2))), \quad \text{(S28)}$$

with $|f(\hat{H}(\mathcal{G}(\lambda, p_1, p_2))) - f(\hat{H}_{\mathrm{Eff}}(\mathcal{G}(\lambda, p_1, p_2)))| = \mathcal{O}(L^{-1/2})$ *for finite* L.

In order to prove this theorem we focus on the two-body graph-dependent operators contained within $\hat{H}(\mathcal{G}(\lambda, p_1, p_2))$. These can be written as

$$\hat{O}(\mathcal{G}(\lambda, p_1, p_2)) = \frac{1}{N_E(\mathcal{G}(\lambda, p_1, p_2))} \sum_{\substack{v,v'=1 \\ v>v'}}^{L} \tilde{p}_{v,v'} \hat{s}_v^\alpha \hat{s}_{v'}^\alpha, \quad \text{(S29)}$$

where $\tilde{p}_{v,v'}$ is a random variable which takes value 1 with probability p_1 if the vertices v and v' belong to the same set of sites, takes value 1 with probability p_2 if they belong to different sets, and takes value 0 otherwise. The quantity $N_E(\mathcal{G}(\lambda, p_1, p_2))$ is the number of edges on the instance of $\mathcal{G}(\lambda, p_1, p_2)$.

Now we will prove the following Lemma

Lemma 5 *Let* $|\psi\rangle$ *be any state such that* $\langle\psi|\psi\rangle = 1$. *Then the following holds*

$$\langle\psi|\hat{O}(\mathcal{G}(\lambda, p_1, p_2))|\psi\rangle = \left\langle \psi \left| \frac{1}{2N_E(\mathcal{G}(\lambda, p_1, p_2))} \left(p_1 \left((\hat{S}_A^\alpha)^2 + (\hat{S}_B^\alpha)^2 \right) + 2 p_2 \hat{S}_A^\alpha \hat{S}_B^\alpha \right) \right| \psi \right\rangle + \mathcal{O}(L^{-1/2}). \quad \text{(S30)}$$

This Lemma will enable us to prove Theorem 4. In order to prove Lemma 5 observe that we can re-write Eq. (S29) as

$$\hat{O}(\mathcal{G}(\lambda, p_1, p_2)) = \frac{1}{N_E(\mathcal{G}(\lambda, p_1, p_2))} \left(\sum_{\substack{v,v'=1 \\ v>v'}}^{\lambda L} p_{v,v'}^1 \hat{s}_v^\alpha \hat{s}_{v'}^\alpha + \sum_{\substack{v,v'=\lambda L+1 \\ v>v'}}^{L} p_{v,v'}^1 \hat{s}_v^\alpha \hat{s}_{v'}^\alpha + \sum_{v=1}^{\lambda L} \sum_{v'=\lambda L+1}^{L} p_{v,v'}^2 \hat{s}_v^\alpha \hat{s}_{v'}^\alpha \right), \quad \text{(S31)}$$

where $p_{v,v'}^1$ and $p_{v,v'}^2$ are random values which take value 1 with probabilities p_1 and p_2 respectively and take value 0 otherwise. The first two summations in the above expression are just two-body operators over an Erdős-Rényi graph of size λL and $(1 - \lambda)L$. We know from Lemma 3 we can replace their expectation with those with their collective counterparts with corrections of order $\mathcal{O}(L^{-1/2})$. In the final summation we can also, up to corrections of order $\mathcal{O}(L^{-1/2})$, replace $p_{v,v'}^2$ with p_2 and move it outside the sum. This again follows from identifying the normal distribution associated with a

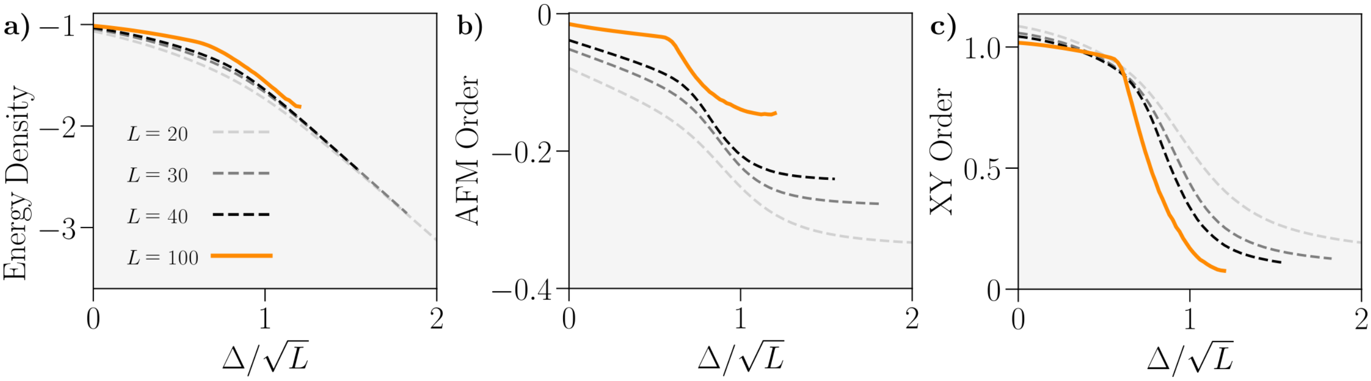

Supplementary Figure 3: Reproduced from the main-text but on a re-scaled x-axis. Properties of the ground-state of the XXZ Hamiltonian on the Erdős-Rényi (ER) graph with $p = 0.5$. **a-c)** Energy density, anti-ferromagnetic order C_{AFM} and XY order C_{XY} versus $\Delta/\sqrt{L}$, with the system size L provided in the legend.

given eigenvalue of the operator and applying a Chernoff and Union bounds to find the most extreme possibilities. From this it follows that Lemma 5 is true.

It is then clear from Weyl's inequality that the largest magnitude eigenvalue of the difference matrix $D = \hat{H}(\mathcal{G}(\lambda, p_1, p_2)) - \hat{H}_{\mathrm{Eff}}(\mathcal{G}(\lambda, p_1, p_2))$ is bounded as $\mathcal{O}(L^{1/2})$. We can now use $N_E(\mathcal{G}(\lambda, p_1, p_2)) = \frac{1}{2}L^2\left(\lambda^2 p_1 + (\lambda - 1)^2 p_1 + 2\lambda(\lambda - 1)p_2\right) + \mathcal{O}(L)$ and invoke Lemma 2 to arrive at the proof of 4.

4.2 Critical Point of the XXZ Limit of $\hat{H}(\mathcal{G}(\lambda, p_1, p_2))$

We now consider the XXZ limit of $\hat{H}(\mathcal{G}(\lambda, p_1, p_2))$ and derive the location of the critical point. Theorem 4 tells us, in the thermodynamic limit, the equilibrium properties of this system are equivalent to those derived from the simpler Hamiltonian

$$\hat{H}_{\mathrm{Eff,XXZ}}(\mathcal{G}(\lambda, p_1, p_2)) = \frac{1}{N}\Bigg(- Jp_1\left(\sum_{\alpha=x,y}(S_A^\alpha)^2 + (S_B^\alpha)^2\right) + \Delta p_1\left((\hat{S}_A^z)^2) + (\hat{S}_B^z)^2\right) - 2Jp_2\left(\sum_{\alpha=x,y}\hat{S}_A^\alpha \hat{S}_B^\alpha\right) + \Delta p_2 \hat{S}_A^z \hat{S}_B^z\Bigg), \tag{S32}$$

for two large spins.

We can minimise this energy with a classical solution where the collective spins A and B are described by vectors (s_A^x, s_A^y, s_A^z) and (s_B^x, s_B^y, s_B^z) with magnitudes $s\lambda L$ and $s(1-\lambda)L$ respectively. The energy is then minimised by polarising both spins A and B in either the $+y$ or $+x$ directions for $\Delta < \Delta_c$ and polarising spins A and B in opposite directions along the z axis for $\Delta > \Delta_c$. Comparing the energies of these states yields the critical point

$$\Delta_c = \frac{p_1(\lambda^2 + (1-\lambda)^2) + p_2\lambda(1-\lambda)}{2p_2\lambda(1-\lambda) - p_1(\lambda^2 + (1-\lambda)^2)} J, \tag{S33}$$

which is valid for any finite s. Substituting the values $\lambda = 1/2$, $p_1 = 1/2$, $p_2 = 1$ and $J = 1$ which we use for Fig. 3 of the main text yields $\Delta_c = 3$ – in agreement with our numerical data.

5 Erdős-Rényi Plots with Re-scaled Axes

Here, in Supplementary Fig. 3, we re-plot the results of Fig. 2 in the main-text — which correspond to the ground-state properties of the spin $s = 1/2$ XXZ model on the Erdős-Rényi graph — but with

the x-axis rescaled by $\sqrt{L}$. We observe that the re-scaling of $\sqrt{L}$ is sufficient to prevent the drift of the critical point seen in the main text. Whilst accurately estimating this critical point is beyond the scope of this work the plots suggests the convergence of critical point to a value in the region $0 < \Delta/\sqrt{L} < 1$.

6 Numerical Details for Matrix Product State Calculations

Here we provide details for the Matrix Product State (MPS) calculations we performed for the XXZ model on various graphs. We utilised the Tensor Network Python (TeNPy) library [6] for our simulations, encoding the initial guess for ground-state as an open-boundary MPS and the Hamiltonian as a long-range Matrix Product Operator (MPO) [7]. Using the Density Matix Renormalisation Group (DMRG) [8] algorithm with single site updates and a mixer enabled we then iteratively improved on our initial guess until convergence was achieved for the specified bond-dimension χ.

We used a direct mapping from the $v = 1..L$ sites of the graph to the sites of the MPS $s = 1...L$. For the graph $\mathcal{G}(\lambda = 1/2, p_1 = 1, p_2 = 1/2)$ we set the first $L/2$ sites of the graph to correspond to one partition and the other $L/2$ to the other (see adjacency matrix in Fig 3 in the main text), no ordering was done beyond this. For the maximally irregular graph we set the site with the highest degree to the central site ($V = L/2$) and have the distance of other sites to the central site increase with decreasing degree (leaving the sites with degrees 1 and 2 as $V = 1$ and $V = L$ respectively). This can be observed in the adjacency matrix in Fig. 4 of the main text. For all other graphs no attempts were made to order the sites of the graph in any specific way and thus the same is true for the sites of the MPS.

Truncation Errors - For a given DMRG run we calculate the total truncation error, i.e. the sum of the squares of all singular values discarded during the final sweep of the routine and in Supplementary Fig. 4 we plot these for the simulations performed in the main text. For random graphs where the ground state is found on multiple instances of the graph and averaging is performed, we plot the average over these instances.

The truncation error is never greater than 1.2×10^{-4}. Combining this with the smoothness of the observables as a function of Δ suggests our results are reasonably accurate and well-converged. The values of χ used for the highest system sizes represent the maximum possible with the computational resources available to us (for $L = 100$ and $\chi = 1600$ we required around 20GB of RAM for each simulation, setting a limit on the available χ).

Notably, we find for the results on the random graphs the truncation error is improved for values of $L \sim 100$ compared to $L \sim 40$ despite using a similar, or even smaller value of χ. We understand this due to finite-size effects (which can be particularly strong due to the statistical fluctuations associated with drawing from a random ensemble) being less significant for the higher value of L. At such higher values of L the results more accurately obey the scaling of the large L solution which, for the dense graphs we study, typically has a bi-partite entanglement entropy that scales as $\log(L)$ and makes the ground-state accessible with a bond-dimension proportional to the system size. We note that such a scaling has previously been observed in the Lipkin-Meshov-Glick model [4] (an all-to-all XY model with a transverse field) and here we observe it for a range of dense graphs.

Energy Convergence - In Supplementary Fig. 4 we also plot examples of the convergence of the ground-state energy obtained versus DMRG sweep number for the range of graphs we consider in the paper and the largest system sizes used. For each graph, we select values of Δ where the truncation errors are highest and show the convergence for values of χ up to that used in our results. All plots show a clear convergence in the ground-state energy with increasing χ and indicate the value of χ used in our work is reasonable, with the energy obtained via DMRG, changing by at most 0.729%

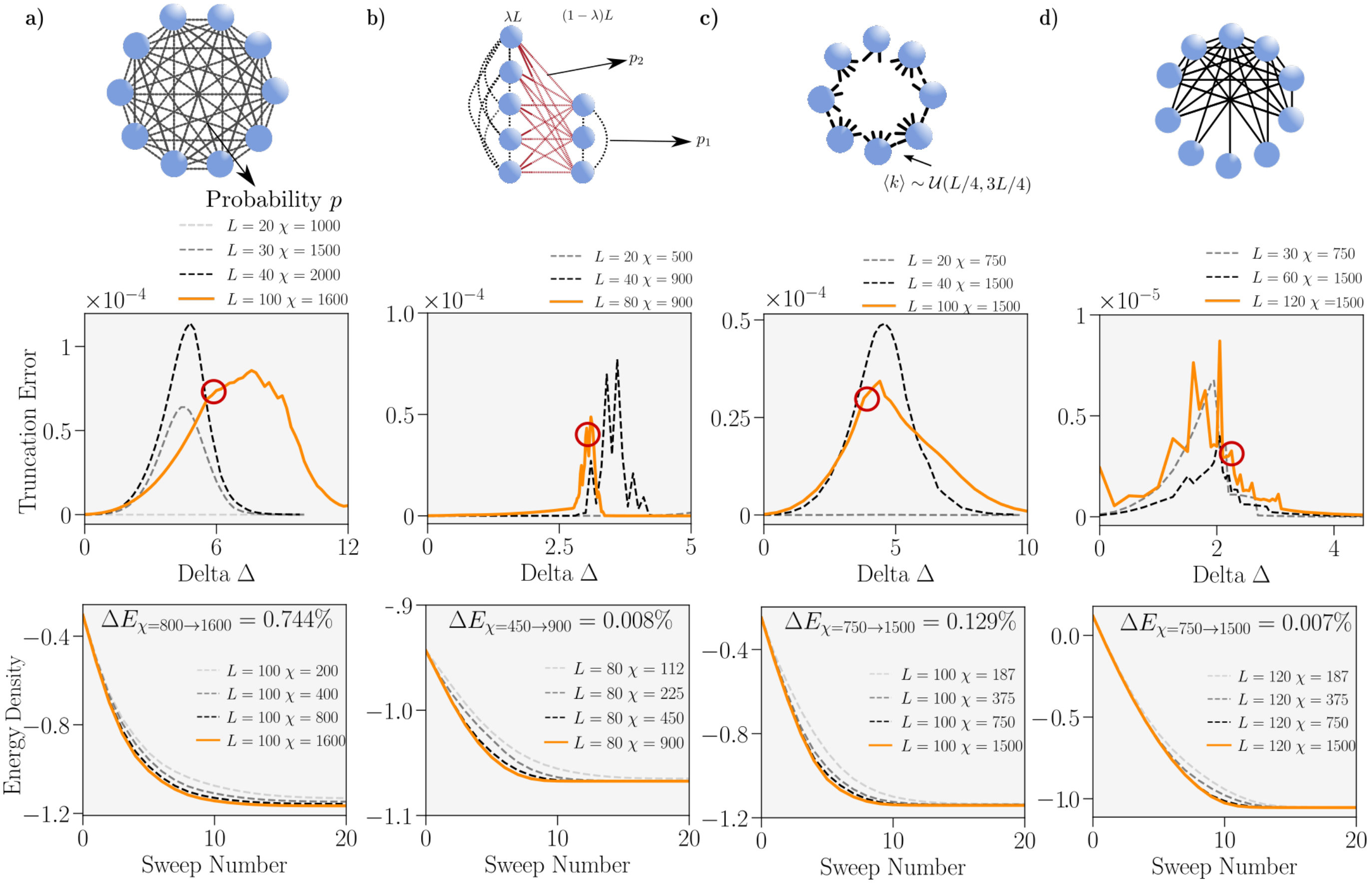

Supplementary Figure 4: Truncation error (total sum of the square of the singular values discarded during the DMRG routine) and energy versus sweep number for the ground-state calculations performed and whose results are provided in the main text.**a-d)** Graphs from Figures 2,3,4 and 5 of the main text respectively with graph schematics provided at the top. Vertically aligned plots correspond to the same graph, which is illustrated at the top along with a legend for the system sizes and bond-dimension used. Upper plots) Truncation error for a range of Δ values. For the graphs drawn from a random ensemble the truncation error is averaged over the same number of draws as in the main text. Lower Plots) Energy Density versus sweep number for the DMRG routine for the highest value of L used and for the Δ values $6.0, 2.9, 4.5$ and 2.3 respectively (marked in red in the plot above). The legend in the plot indicates the bond-dimensions used and we also annotate the percentage change in energy density ΔE when doubling the bond-dimension to reach the value used in our work. For the graphs drawn from a random ensemble the plots correspond to a single instance which was selected in a completely unbiased manner.

(and generally by over an order of magnitude less) when we double the bond-dimension to reach the value used in our results.

Calculation of the Shannon Entropy for various graphs - In the main text we also calculated, for the maximally irregular graph, the Shannon entropy which is defined as

$$H(\langle \hat{\sigma}_v^\alpha \hat{\sigma}_{v'}^\alpha \rangle) = \sum_{i=0}^{n-1} p_i \log_2(p_i), \tag{S34}$$

where p_i is the fraction of elements of the off-diagonal $L \times L$ matrix $\langle \hat{\sigma}_v^\alpha \hat{\sigma}_{v'}^\alpha \rangle$ whose values are in the range $[-1 + 2i/n, -1 + 2(i+1)/n]$. Here, in Supplementary Fig. 5, we plot this quantity (setting $n = 256$) for all the graphs considered in the main text (aside from the maximally irregular which is already plotted in Fig. 5 of the main text) and the 1D chain. We compute the entropy along both the $\alpha = x$ and $\alpha = z$ spin axes.

In the Erdős-Rényi graph we find the entropy in both spin axes is maximised at the critical point

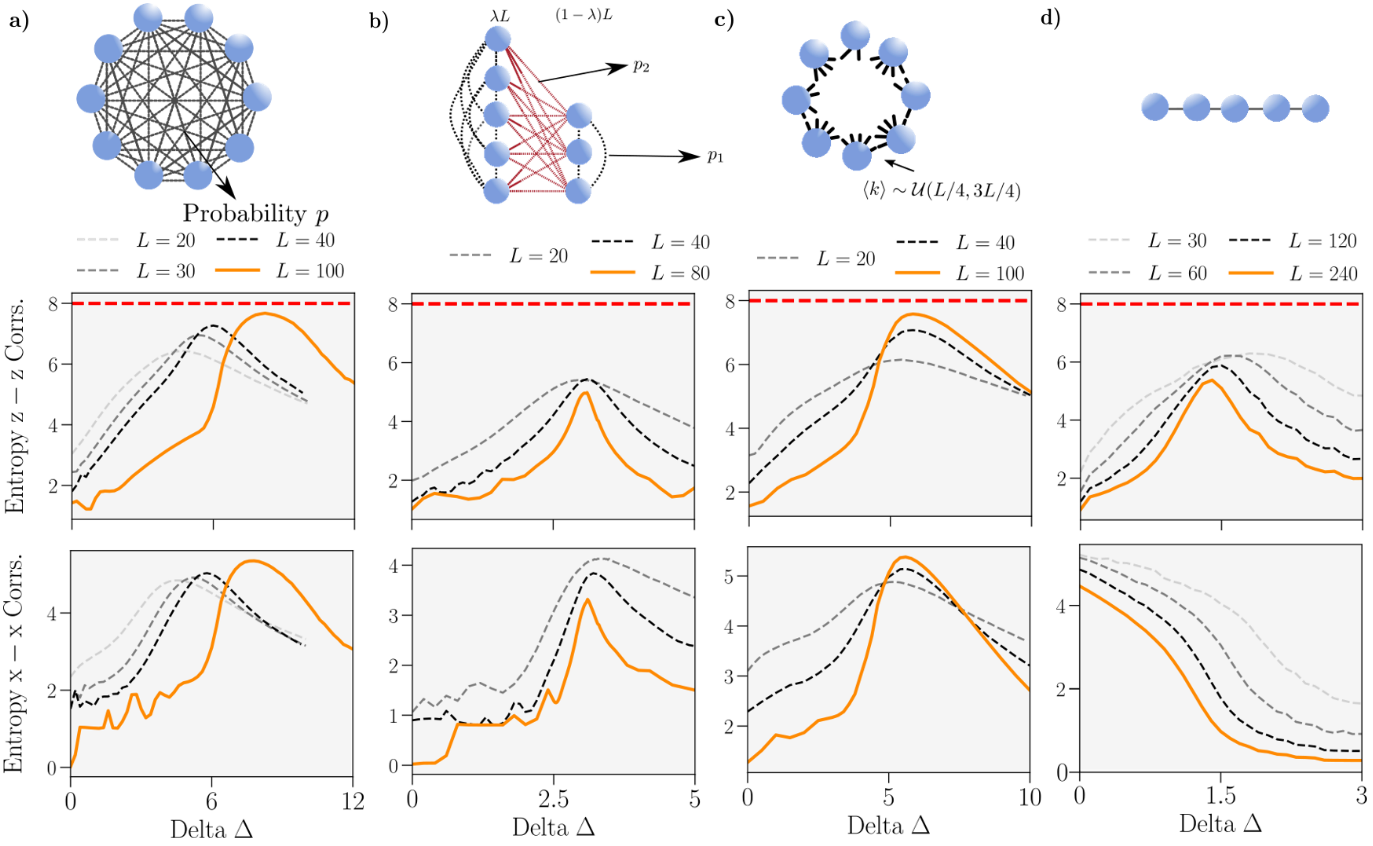

Supplementary Figure 5: Shannon Entropy — see Eq. (S34) — of the off-diagonal correlations in the ground-state of the XXZ model versus Δ for various different graphs and system sizes. We use $n = 256$ bins for the off-diagonal correlations. **a-d)** Graphs used in Figures 2, 3, 4 of the main text and the 1D Chain respectively. Upper plots are the entropy $H(\langle \hat{\sigma}^z_v \hat{\sigma}^z_{v'} \rangle)$ whilst the lower are $H(\langle \hat{\sigma}^x_v \hat{\sigma}^x_{v'} \rangle)$. Red-dotted line indicates the maximum possible Shannon Entropy for the $n = 256$ bins used. Above the plot we provide illustrations of the respective graphs and colour code for the system sizes. We adopt the same averaging over random instances of the graphs as in the main text and the same bond dimensions as specified in Supplementary Fig. 4. No averaging is required for the chain as it is unique for a given L. The bond dimension used for the chain is $\chi = 500$ for all system sizes.

and this maximum is not diminishing. We know, however, that the critical point diverges with L and the ground state is always the Dicke-state $|L/2, M\rangle$ for finite Δ as $L \to \infty$. The Shannon entropies $H(\langle \hat{\sigma}^x_v \hat{\sigma}^x_{v'} \rangle)$ and $H(\langle \hat{\sigma}^y_v \hat{\sigma}^y_{v'} \rangle)$ are both zero for this state as all off-diagonal correlations take the same value. Both entropies will thus be 0 on the ER graph for all finite Δ as $L \to \infty$. For the random graph with a non-trivial cut, i.e. $\mathcal{G}(\lambda, p_1, p_2)$ we find the entropy in both axes is diminishing with system sizes and far from it's maximal value. Moreover, from our solution in Section 3 we know that for $L \to \infty$ the system's ground state is the Dicke state for $\Delta < \Delta_c$ and an anti-ferromagnet for $\Delta > \Delta_c$. The entropy of the $x - x$ correlations will thus always be 0 and the entropy of the $z - z$ correlations will be 0 for $\Delta < \Delta_c$ and $\lambda \log_2(\lambda) + (1-\lambda)\log_2(1-\lambda)$ for $\Delta > \Delta_c$, which has an upper bound of 1. For the irregular dense graph with $k \sim \mathcal{U}(L/4, 3L/4)$ we find qualtitatively the same features as the maximally irregular graph in the main text: the entropy in both spin axes is maximal at the critical point and not diminishing with system size. For the 1D chain we find the entropy in both spin axes is diminishing with system size. Moreover, because there is no long-range $x - x$ order in the ground-state in the thermodynamic limit we know the $x - x$ entropy must vanish as measuring $\langle \hat{\sigma}^x_v \hat{\sigma}^x_{v'} \rangle$ for a random pair of sites as $L \to \infty$ will yield 0 with probability tending to 1.

7 Transverse Field Ising Model

To supplement our numerical results for the XXZ limit of the Hamiltonian in Eq. (S1) we have also performed numerical calculations for the transverse field Ising (TFI) limit of Eq. (S1). Specifically, taking $s = 1/2$, $J_y = J_x = w_z = w_y = 0$, $J_z = -1$, $w_x = h$ we consider (for conciseness we have scaled the terms in Eq. (S1) – none of the Physics is changed) the Hamiltonian for the total energy:

$$\hat{H}_{\text{TFI}}(\mathcal{G}) = -\frac{L}{N_E} \sum_{(v,v') \in E} \hat{\sigma}^z_v \hat{\sigma}^z_{v'} + h \sum_{v \in V} \hat{\sigma}^x_v. \tag{S35}$$

In Supplementary Fig. 6 we use Matrix Product State simulations to compare results from the ground state of this Hamiltonian on the maximally irregular graph to results for the ground state properties on the ER graph. Our finite-size numerics for the ER graph demonstrate convergence to exact results for $L = \infty$, which correspond to the well-known solution to the TFI model on the complete graph [9]. The second order phase transition which is manifest on the ER graph is thus directly underpinned by the system behaving like a single collective spin – the randomness and inhomogeneity inherent in the actual adjacency matrix plays no role in the Physics of the system. This is further evidenced by the diminishing of the Shannon entropy of the two-point correlations with increased system size.

Whilst a second order transition between ferromagnetic and paramagnetic phases is also manifest in the ground-state on the maximally irregular graph the functional behaviour of observables in the system is distinct from the ER graph. Moreover, the Shannon entropy of the two point correlations is not diminishing with system size, significant along all spin axes, maximised near the critical point and occurs in unison with a non-zero entanglement entropy in the system. These results serve as further numerical evidence of Theorem 1 and reinforce our conclusions from the main text, emphasizing that they are not specific to the XXZ limit of $\hat{H}(\mathcal{G})$.

We note that we added a small term of the form $\frac{1}{200L} \sum_{v \in V} \hat{\sigma}^z$ to Eq. (S35) when performing the DMRG simulations to break the Z_2 symmetry and ensure the longitudinal magnetisation and entanglement entropy are well-defined — all other physical observables are affected negligibly by this term. We also assume such a term, which is negligible other than breaking the Z_2 symmetry and forcing $\langle \hat{S}^z \rangle \geq 0$, has been added to the Hamiltonian in the ER case for $L = \infty$ in order to calculate the longitudinal magnetisation density.

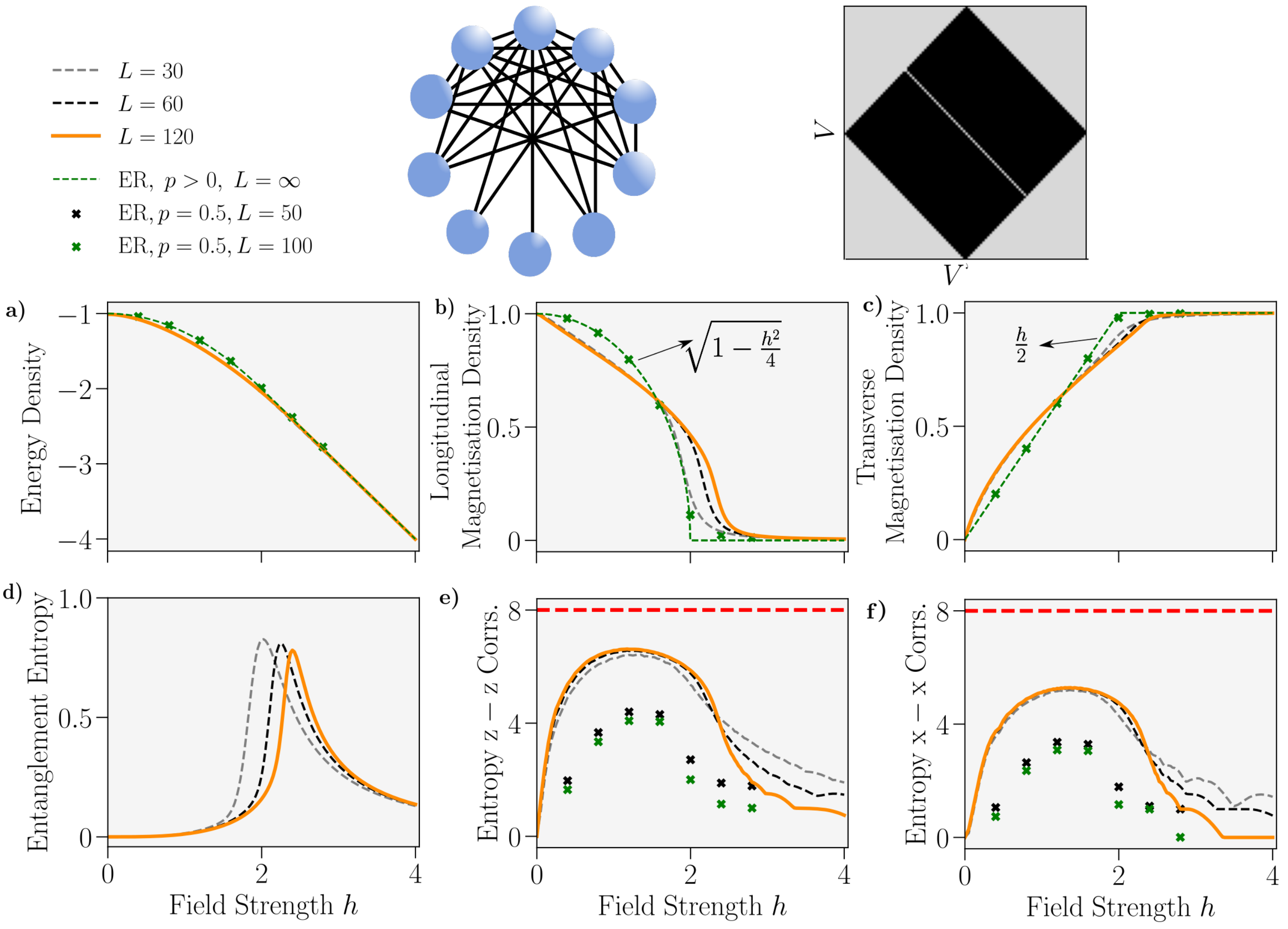

Supplementary Figure 6: Properties of the ground-state of the spin-$\frac{1}{2}$ TFI Hamiltonian – see Eq. (S35) – on the 'Maximally Irregular Graph' where each pair of vertices, other than two, have a different degree. Graph schematic alongside adjacency matrix for $L = 100$ is provided. System sizes are coded by colour and a full legend is provided top left. **a-f)** Ground-state energy density, longitudinal magnetisation density $\langle \hat{S}^z \rangle / L$, transverse magnetisation density $\langle \hat{S}^x \rangle / L$, von-Neumann entanglement entropy between sites $V = 1 ... L/2$ and $V = L/2 + 1 ... L$ and Shannon-Entropy of the $z - z$ and $x - x$ correlations (see Methods) versus transverse field strength h. We use $n = 256$ bins to calculate the Shannon Entropy. A dotted red line has been added to plots e) and f) to indicate the maximum possible value for the Shannon Entropy with the numbers of bins used. Bond dimensions of $\chi = 250, 500$ and 500 have been used for system sizes $L = 30, 60$ and 120 respectively. For comparison, results have been added for the ER graph: the green dashed line represents the exact expression for ground-state observables of $\hat{H}_{\rm TFI}(\mathcal{G}_{\rm ER}(p))$ for any finite non-zero p in the thermodynamic limit and the black and green markers represent DMRG calculations for the ER graph with $p = 0.5$ and $L = 50$ and $L = 100$ respectively. The finite-size ER results were averaged over $n = 10$ draws of the ER graph from its ensemble. A bond dimension of $\chi = 500$ was used for all finite-size calculations — which we found sufficient to reach our desired accuracy.

References

[1] M. Krivelevich and B. Sudakov, *Pseudo-random Graphs*, pp. 199–262. Berlin, Heidelberg: Springer Berlin Heidelberg, 2006.

[2] A. Bertoni, P. Campadelli, and R. Posenato, *An upper bound for the maximum cut mean value*, pp. 78–84. 1997.

[3] H. Chernoff, "A Measure of Asymptotic Efficiency for Tests of a Hypothesis Based on the sum of Observations," *The Annals of Mathematical Statistics*, vol. 23, no. 4, pp. 493 – 507, 1952.

[4] J. I. Latorre, R. Orús, E. Rico, and J. Vidal, "Entanglement entropy in the Lipkin-Meshkov-Glick model," *Phys. Rev. A*, vol. 71, p. 064101, 2005.

[5] J. Tindall, F. Schlawin, M. A. Sentef, and D. Jaksch, "Analytical solution for the steady states of the driven Hubbard model," *Phys. Rev. B*, vol. 103, p. 035146, Jan 2021.

[6] J. Hauschild and F. Pollmann, "Efficient numerical simulations with Tensor Networks: Tensor Network Python (TeNPy)," *SciPost Phys. Lect. Notes*, 2018. Code available from https://github.com/tenpy/tenpy.

[7] C. Hubig, I. P. McCulloch, and U. Schollwöck, "Generic construction of efficient matrix product operators," *Phys. Rev. B*, vol. 95, p. 035129, 2017.

[8] S. R. White, "Density matrix formulation for quantum renormalization groups," *Phys. Rev. Lett.*, vol. 69, pp. 2863–2866, 1992.

[9] J. Strecka and M. Jascur, "A brief account of the Ising and Ising-like models: Mean-field, effective-field and exact results," 2015.
